# Central and peripheral relationships between morphine and glucose on antinociception in rats

**Published:** 2014

**Authors:** Rinah T. Yamamoto, Robin B. Kanarek

**Affiliations:** Department of Psychology, Tufts University, Medford, MA 02155, USA

**Keywords:** Antinociception, glucose, morphine, periaqueductal gray

## Abstract

Previous research from our laboratory has determined that in the absence of a gustatory response or taste hedonics, intraperitoneal (i.p.) glucose administration enhanced morphine-mediated analgesia in rats and had antinociceptive actions on its own. Two experiments examined the potential of a central mechanism for glucose’s actions on morphine-mediated antinociception. Morphine (2.5 µg) was infused into the periaqueductal gray (PAG) while glucose (300 mg/kg) was injected into the peritoneal cavity, or glucose (32 nmol) was infused into the PAG while morphine (3.2 mg/kg) was injected i.p. Doses of morphine and glucose were selected based on our own prior research for being below the threshold for analgesic efficacy. Antinociception was assessed using the hot-water tail-withdrawal procedure. Tail-withdrawal latency was tested at baseline (before), and 12, 24 and 36 minutes after the i.p. injection. The results indicated that 300 mg/kg glucose, administered i.p. effectively increased the antinociceptive potency of a low dose of centrally administered morphine, while central infusion of glucose enhanced peripheral morphine-mediated antinociception. These outcomes support previous evidence of glucose’s influence on the antinociception actions of opioid drugs. Furthermore, they suggest that glucose produces its enhancing actions on morphine-mediated antinociception in the brain. These results support the hypothesis that glucose does not need to go through a gustatory mechanism or taste hedonics to alter morphine’s antinociceptive actions.

## Introduction

Intake of palatable sweet-tasting substances, such as sucrose and glucose solutions, can alter behavioral and physiological responses to opioid drugs in animals [1–3]. However, the specific effect of sweet tasting substances on these responses is not consistent, but rather varies as a function of the nutritive value of the palatable food or fluid. For example, intake of nutritive sweet-tasting solutions enhances morphine-mediated antinociception (MMA) while intake of non-nutritive sweet-tasting solutions is without effect [4–6]. Furthermore, acute consumption of a sucrose solution alone produces antinociceptive behavior that is reduced in the presence of μ opiate receptor antagonists, suggesting involvement of the endogenous opioid system [3,7,8].

An important question to be addressed is the mechanism underlying the effects of nutritive sweet-tasting solutions on the actions of opiate drugs. Some researchers maintain that sweet substances alter MMA through taste hedonics and gustatory signals that are transmitted to the central nervous system [9–13]. However, sweet substances might also have more general physiological consequences. One way to determine if the effects of intake of sweet substances are due to their hedonic properties or reflect a more systemic action would be to bypass gustatory responses by administering a sweet solution directly into the peritoneal cavity. Research from our laboratory has shown that an intraperitoneal injection of glucose, which serves as a primary energy source for metabolic processes in both the periphery and central nervous system, enhances the antinociceptive properties of the μ-opioid agonist, morphine [14]. Moreover, intraperitoneal glucose administration, on its own, produces antinociceptive actions in rats that can be measured using the hot-water tail-withdrawal procedure [14]. These results imply that the alteration in MMA seen in rats consuming palatable solutions is not simply a consequence of the hedonic properties of the solution [15].

In two pilot studies, a low dose of morphine was administered into either, the lateral ventricles (i.c.v.) or peritoneal cavity (i.p.) of rats, while a low dose of glucose was administered into the peritoneal cavity or lateral ventricles, respectively. In both pilot studies, glucose and morphine at doses that did not produce antinociception on their own, produced a significant increase in tail withdrawal latency in the hot water tail withdrawal test when combined [16].

To further investigate whether the antinociceptive actions of glucose are mediated within the central nervous system, low doses of morphine (or glucose) were infused into the PAG of rats concomitant with low doses of glucose (or morphine) injected into the peritoneal cavity followed by an assessment of morphine-mediated antinociception. The PAG is associated with both opiates and pain. It is not only an important brain-site relaying nociceptive information but also has a high density of μ-opiate receptors [17–21]. We hypothesized that administration of low doses of morphine and glucose alone would not produce antinociception while central and peripheral co-administration of glucose and morphine would produce antinociception. If, at low doses, peripheral or central glucose administration potentiates the antinociceptive actions of a central or peripheral morphine injection, respectively, this would provide evidence that glucose is altering MMA through a central mechanism.

## Methods

### Animals

Adult male Long-Evans rats (Charles River Breeding Laboratories, Portage, MI) weighing 250–275 g at the start of the experiment were used. Rats were housed individually in standard stainless-steel cages in a temperature-controlled room (22±1°C) maintained on a reverse 12–12 hr light-dark cycle (lights on: 2000-0800 hr). Rats had unrestricted access to standard Purina Rodent Pellets (#5001) and water. All rats were allowed to acclimate to the laboratory and handling procedures for at least one week prior to the initiation of experimental procedures. Rats were handled daily by the same experimenter to reduce the possibility of stress-related behaviors during antinociceptive testing. All testing took place during the dark phase of the 24-hour cycle.

These studies were carried out in accordance with the NIH Guidelines for the Care of Laboratory Animals. The Tufts University Institutional Animal Care and Use Committee approved all of the experimental protocols. All efforts were made to minimize animal suffering and to keep the number of animals used to the minimum necessary.

### Surgery

Surgery was performed at least one week after the rats were delivered to the laboratory. Rats were anesthetized with 6 mg/kg xylazine in combination with 100 mg/kg ketamine administered i.p. Using a stereotaxic apparatus, stainless steel guide cannulae (Plastics One, Roanoke, VA) were implanted into the PAG (from bregma −7.6 AP, −0.8 ML and −5.8 DV) [22]. Dummy cannulae were inserted to maintain cannulae guide integrity. Immediately following surgery, rats were administered 20 mg/kg cefazolin antibiotic, intramuscularly, and allowed to recover in their home cages for a minimum of one week prior to antinociceptive testing.

### Drugs

For peripheral administration, morphine sulfate (generously donated by the National Institute on Drug Abuse) was dissolved in 0.9% saline to a concentration of 3.2 mg/ml and administered i.p. in a volume of 1.0 ml/kg. For PAG administration, 2.5 µg morphine sulfate was dissolved in 0.5 µl 0.9% saline.

For peripheral administration, D-glucose (ICN Biomedicals) was dissolved in sterile water at a concentration of 300 mg/ml (1.665 molar concentration), and administered i.p. in a volume of 1.0 ml/kg. For PAG administration, D-glucose was dissolved in sterile water to produce 32 nmol. While there is some inconsistency in the literature on the morphine dose threshold for antinociception, the morphine and glucose doses used in the current studies were selected based on research on MMA [23–30] and by our own prior research on both MMA and glucose-mediated antinociception [14].

Morphine and glucose, or their respective vehicles, were infused into the PAG through a 28-gauge internal injection cannula extending 0.1 mm beyond the guide cannula. The internal cannula was connected using PVC (#32) tubing filled with morphine, glucose, physiological saline or sterile water and infused using a gas tight 25 µg Hamilton syringe and a Harvard infusion pump. The infusion volume was 0.5 µl for PAG injections delivered over one minute. Cannulae were left in situ for 30 s following drug infusions.

### Determination of antinociceptive responses

Each rat was gently held in a clean towel and placed on a platform, level with the top of a hot-water bath maintained at 54±0.2°C. The rat’s tail was gently lowered 4 cm into the hot water bath. The behavioral response criterion was removal of the tail from the hot water bath. To prevent tissue damage if a rat failed to remove its tail within 12 s, the tail was gently removed from the water bath by the experimenter [31]. Tail withdrawal latency was measured to the nearest 0.1 second using a stopwatch. Antinociceptive testing was performed prior to drug administration (baseline) and at 12, 24, and 36 minutes after drug injections.

### Effects of morphine administration into the PAG and intraperitoneal glucose injections on antinociceptive responses

Following determination of baseline tail-withdrawal latencies, drug-naive rats were infused with morphine or saline into the PAG followed immediately by i.p. injections of glucose or sterile water. Eight rats received injections of 2.5 µg morphine PAG+300 mg/kg glucose i.p. eight rats, 2.5 µg morphine PAG+sterile water i.p. eight rats, saline PAG+300 mg/kg glucose i.p. and eight rats, saline PAG+sterile water i.p.

### Effects of glucose administration into the PAG and intraperitoneal morphine injections on antinociceptive responses

Following determination of baseline tail-withdrawal latencies, drug-naive rats were infused with glucose or sterile water in the PAG followed immediately by i.p. morphine or saline injections. Eight rats received injections of 32 nmol glucose into the PAG+3.2 mg/kg morphine i.p. eight rats, sterile water into the PAG+3.2 mg/kg morphine i.p. seven rats, 32 nmol glucose into the PAG+saline i.p. and seven rats, sterile water into the PAG+saline, i.p.

### Histology

After the conclusion of each experiment, rats were euthanized with carbon dioxide and the brains removed immediately and placed in a 4% paraformaldahyde solution. Brains were subsequently sectioned into 40-micron slices, stained with cresyl violet and assessed for cannulae placement. Rats with incorrect cannulae placements were removed from analyses.

### Statistical analysis

Antinociceptive responses are expressed as percent maximal possible effect (%MPE) using the formula (Dewey and Harris, 1975):

$$\%MPE=\frac{\text{test latency}-\text{baseline latency}}{\text{maximal latency}(12s)-\text{baseline latency}}\times 100$$

Repeated-measures ANOVAs were used to analyze %MPEs across time, using morphine and glucose doses as between-subjects factors and time after glucose administration as the within-subjects factor. Post-hoc comparisons were assessed with Bonferroni t-test to assess group differences.

## Results

### Effects of an injection of 2.5 µg morphine into the PAG with an intraperitoneal injection of 300 mg/kg glucose on antinociceptive responses

Histology confirmed that cannulae were in the PAG in all but four rats, which were removed from the data.

There were no significant differences in baseline tail-withdrawal latencies among the groups prior to drug injections (mean±standard deviation (sd): PAG saline with i.p. sterile water 3.3±1.0 sec, PAG saline with i.p. glucose 3.3±0.6 sec, PAG morphine with i.p. sterile water 3.5±0.7 sec and PAG morphine with i.p. glucose 2.7±0.4 sec).

**Figure 1:**
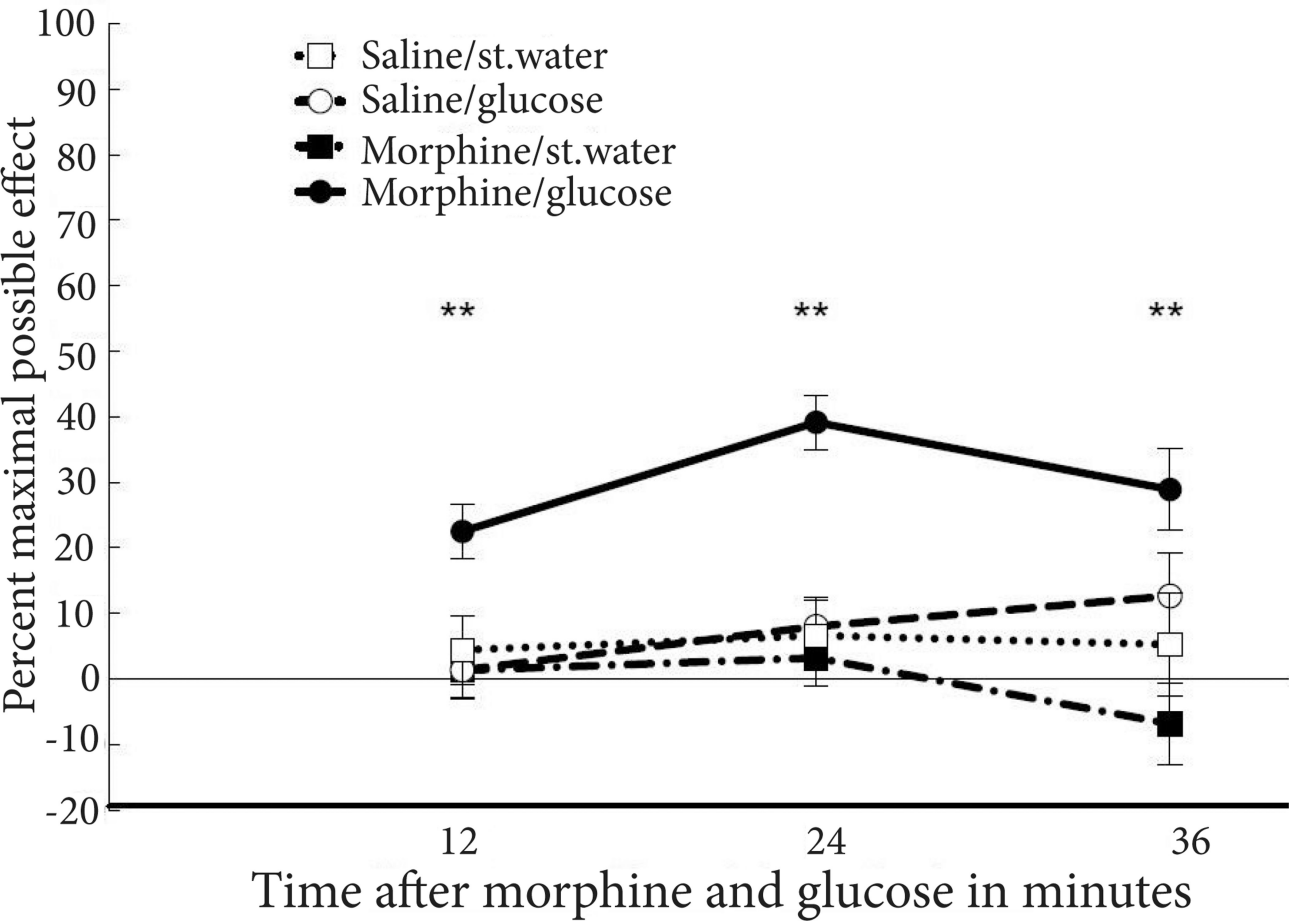
Antinociceptive responses on the hot water tail withdrawal test in rats following injections of 2.5 µg morphine or saline into the PAG and i.p. injections of 300 mg/kg glucose or sterile water. Glucose significantly enhanced antinociceptive responding following administration of morphine into the PAG relative to rats in the other drug conditions. Values represent percent maximal possible effect with error bars representing standard error of the mean. Significant differences at p<0.01 are marked with a double asterisk (**).

Across the three measurement times, antinociceptive responses of rats that received an injection of 2.5 µg morphine into the PAG and i.p. injection of 300 mg/kg glucose were significantly greater than those of animals in all other drug combinations (F_3,24_=1.83, p<0.0001; partial η^2^=0.6). Post-hoc Bonferroni t-tests revealed that %MPEs were significantly greater for rats receiving an injection of 2.5 µg morphine into the PAG and i.p. injection of 300 mg/kg glucose than for rats that received an injection of 2.5 µg morphine into the PAG and i.p. injection of sterile water, or an injection of saline into the PAG and an i.p. injection or either glucose or saline. The lack of efficacy of the morphine infusion on its own or the glucose injection alone confirmed that these doses were below the analgesic thresholds for these substances and is consistent with our prior research [14]. Additionally, one-way ANOVAs demonstrated that at 12, 24 and 36 minutes after injections, %MPEs for rats co-administered morphine into the PAG and glucose into the peritoneal cavity were significantly greater than %MPEs for rats receiving any other drug combination (%MPE 12 min, F_3,24_=5.77; %MPE 24 min, F_3,24_=14.99; %MPE 36 min, F_3,24_=5.67; all p values<0.01) (Figure 1).

### Effects of an injection of 32 nmol glucose into the PAG with an intraperitoneal injection of 3.2 mg/kg morphine on antinociceptive responses

Histology confirmed that cannulae were located in the PAG in all but three rats, which were removed from the data analyses.

There were no significant differences in baseline tail-withdrawal latencies between the groups that subsequently received injections of 32 nmol glucose or sterile water into the PAG and i.p. injections of either 3.2 mg/kg morphine or saline (mean±sd: PAG sterile water with i.p. saline 3.2±0.7 sec, PAG sterile water with i.p. morphine 3.4±0.6 sec, PAG glucose with i.p. saline 2.8±0.6 sec and PAG glucose with i.p. morphine 3.3±0.7 sec).

**Figure 2:**
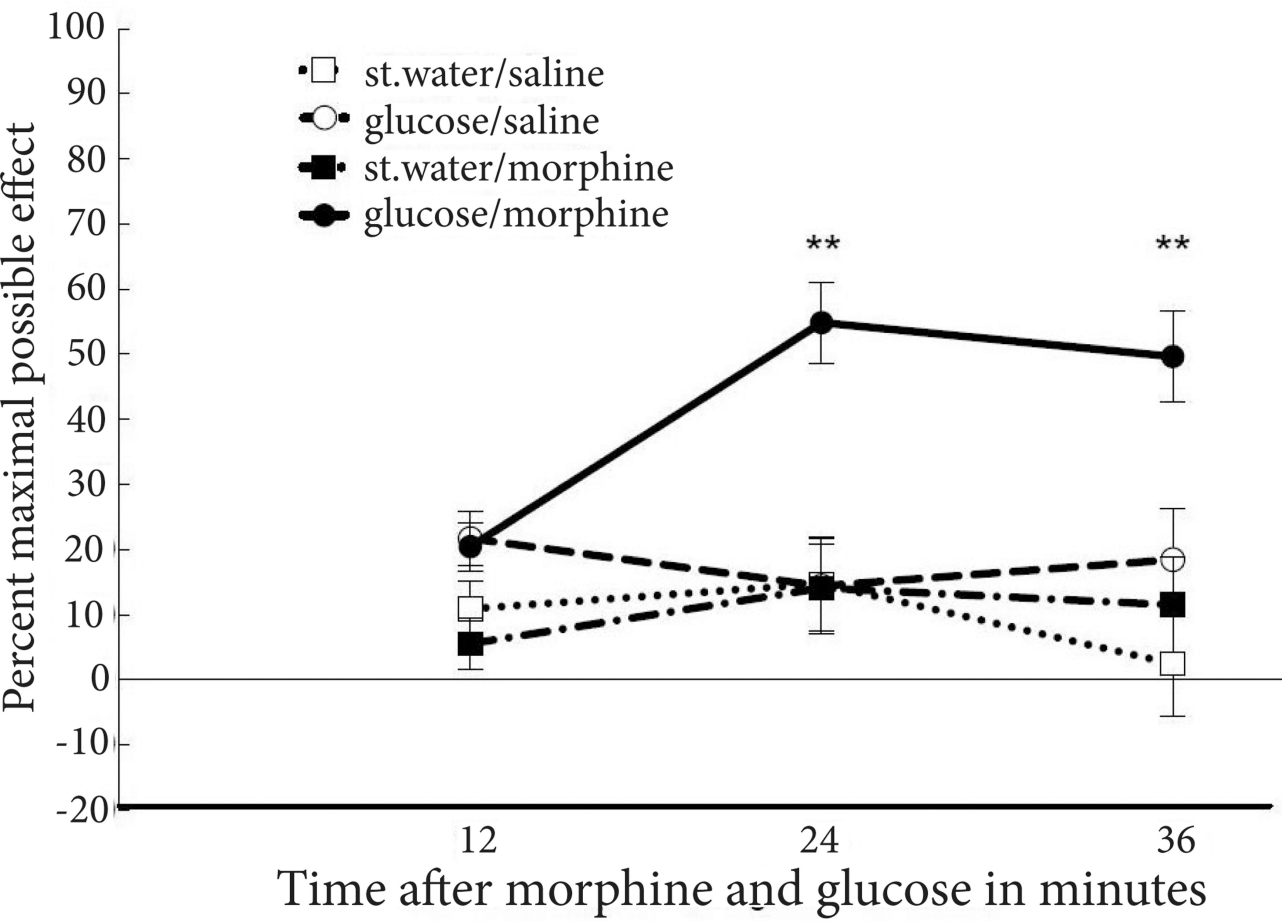
Antinociceptive responses in rats on the hot water tail withdrawal test following administration of 32 nmol glucose into the PAG or sterile water and i.p. injections of 3.2 mg/kg morphine or saline. Glucose in the PAG significantly enhanced antinociceptive responding following i.p. injections of morphine, relative to rats in the other drug conditions. Values represent percent maximal possible effect with error bars representing standard error of the mean. Significant differences at p<0.01 are marked with a double asterisk (**).

Repeated-measures ANOVA determined that there was a significant group by time interaction (F_6,46_=3.21, p<0.01; partial η^2^=0.3) (Figure 2). There was a significant main effect for drug group (F_3,23_=15.08, p<0.0001; partial η^2^=0.7). Post-hoc Bonferroni t-tests indicated that the 32 nmol glucose (PAG) and 3.2 mg/kg morphine (i.p.) combination increased %MPEs in rats relative to all other drug combinations (all p values<0.003). One-way ANOVAs demonstrated that at 12, 24 and 36 minutes after injections, %MPEs for rats co-administered glucose into the PAG and morphine into the peritoneal cavity were significantly greater than %MPEs for rats receiving any other drug combination (%MPE 12 min, F_3,23_=3.74, p<0.025; %MPE 24 min, F_3,23_=9.78, p<0.0001; %MPE 36 min, F_3,23_=8.09, p<0.001).

## Discussion

These experiments demonstrate that in the absence of gustatory input, glucose modulates the antinociceptive actions of both centrally and peripherally administered morphine in rats. A low dose of morphine (2.5 µg) infused into the PAG or administered peripherally (3.2 mg/kg i.p.) was not sufficient on its own to produce an antinociceptive response. However, a significant antinociceptive response was observed when glucose (300 mg/kg i.p. or 32 nmol PAG) was co-administered with morphine. These results support our preliminary results and validate our hypotheses. To our knowledge, this is the first report of this effect.

Numerous studies have examined the effects of palatable sweet substances on the analgesic actions of opiate receptor agonists such as morphine [6,32–34]. Findings from these studies show that intake of sweet substances enhances morphine-mediated MMA. It has been suggested that palatability and hedonics play a role in the ability of sweet substances to alter morphine-mediated antinociception [10–13,35]. This study circumvented the effects of palatability and hedonics by administering glucose directly into the peritoneal cavity or into the PAG, a region rich in opiate receptors. Thus our results imply that glucose influences antinociceptive activity, independent of its palatability and hedonic value.

Mechanisms that could account for the present observations include glucose modulation of receptor binding, endogenous opioid levels, or receptor affinity. Rats consuming sugar solutions demonstrate increased opiate receptor binding [5,36–38]. Further, sucrose ingestion has been linked to increased **β**-endorphin release [39,40] and enhanced c-Fos immunoreactive response, a marker of neuronal excitation, to naloxone [13], an opiate receptor antagonist. Sucrose also inhibits opioid receptor internalization, which would increase the number of available receptors, thus reducing the concentration of morphine needed to produce an effect [41–43].

In the present studies, co-administration of very low antinociceptive doses of morphine and glucose, via combinations of central and peripheral routes, produced antinociceptive behavioral responses. This confirms prior data that includes evidence that i.p. glucose has antinociceptive actions on its own [14]. In that study, we did not note a dose-response profile for glucose on antinociception. A review of the literature to date has shown that ours is the only laboratory reporting research on the analgesic properties of glucose by itself. In addition to our own laboratory, other researchers have investigated antinociceptive behaviors relating to combinations of sucrose (feeding) and MMA [for examples see; 44–47].
